# Impact of COVID-19 status on patients receiving neuraxial analgesia during labor: A national retrospective-controlled study

**DOI:** 10.5339/qmj.2022.30

**Published:** 2022-07-11

**Authors:** Eynas Omer Ibrahim Abdalla, Seema Nahid, Sikha Shastham Valappil, Srinivas Gudavalli, Soumaya Sellami, Noureddine Korichi, Shamsa Ahmad, Victor Vicente Canizares Cespedes, Santhosh Gopalakrishnan

**Affiliations:** Anesthesia Department, Hamad Medical Corporation, Doha, Qatar E-mail: eabdalla1@hamad.qa

**Keywords:** Coronaviruses, COVID-19, Pregnancy, Normal delivery, Neuraxial labor analgesia

## Abstract

Background: Pregnancy affects a woman's susceptibility to and severity of certain infectious diseases. Central neuraxial block for analgesia during labor is superior to nonneuraxial methods in efficacy, safety, and maternal satisfaction. Although Coronavirus disease (COVID-19) can be vertically transmitted from mother to fetus, little is known about the effects of COVID-19 on pregnant women or about anesthesia management and the risk of adverse effects related to neuraxial techniques in women with untreated COVID-19 during gestation.

Aim: This investigation assesses the effects of neuraxial analgesia during labor of COVID-19-positive parturients on their hemodynamic stability.

Results: The study was conducted on 64 patients and involved 32 parturients positive for SARS-CoV-2 by polymerase chain reaction (PCR) and a similar number of control “negative” patients. The affected group had an uneventful course during gestation. Seven were positive for ground-glass opacities on chest X-rays, and none underwent computed tomography (CT) scans. Two neonates were PCR-positive for SARS-CoV-2, and all 32 neonates were released from the hospital. No clinical differences were observed between the neonates in the COVID-19 and control groups. Although parturients in both groups were hemodynamically stable, hemodynamic stability was subnormal in the COVID-19 group regarding blood pressure, oxygen saturation, heart rate, and body temperature. None of the women in either group required a vasopressor or oxygen supplementation during delivery. No other clinical differences were observed between the COVID-19 and control groups.

Conclusion: This is the first case-controlled study testing the anesthetic implications of neuraxial labor analgesia in pregnant, COVID-19-positive women. Although management of neuraxial labor analgesia did not differ in pregnant women positive and negative for COVID-19, their hemodynamic characteristics differed significantly. Therefore, care is required to prevent adverse outcomes in pregnant women positive for COVID-19.

## Introduction

Coronaviruses (CoVs) are a family of single-stranded ribonucleic acid (RNA) viruses that are widely present in bats, birds, and other mammals, including humans.^1^ Coronavirus disease (COVID-19) is a highly contagious disease caused by a recently identified CoV, SARS-CoV-2, first detected in Wuhan, China, in December 2019. CoVs cause respiratory infections in humans, including severe acute respiratory syndrome (SARS) and the Middle East respiratory syndrome, which vary in intensity from mild to severe. In March 2020, the World Health Organization announced that COVID-19 was a global pandemic affecting many countries.^2^ Most studies to date have evaluated the effects of COVID-19 on general populations,^3,4^ with fewer known effects on susceptible groups, such as parturients. However, the physiological changes during pregnancy may increase the risk of infection and/or exacerbate its intensity compared with the general population.^5^ Hence, it is a vulnerable group to study.

Neuraxial labor analgesia (NLA) is the most effective analgesic technique associated with maternal and neonatal safety during labor. Early epidural administration can avoid respiratory symptoms worsening during labor and reduce the need for aerosol-generating procedures, such as general anesthesia if an emergency cesarean section is required in COVID-19 patients. NLA in COVID-19 positive parturients can be hazardous given the multiple abnormalities, such as thrombocytopenia, reported in about one-third of COVID-19 patients, and coagulopathy as a result of a prothrombotic sequence of the underlying infection.^1,6^ The possibility of viremic blood seeding the epidural space theoretically can result in meningitis among COVID-19 positive patients.^7^ Furthermore, even the most experienced medical professionals encounter technical difficulties and have significant challenges introduced when wearing personal protective equipment.

Among the advantages of NLA in COVID-19 patients is preventing respiratory deterioration during labor and delivery. The epidural catheter can be used for anesthetic administration in patients requiring an emergency cesarean section. The sympathetic blockade induced by neuraxial anesthesia can provoke maternal hypotension, which has a potential risk for transient tachypnea in newborns. Several studies have examined hypotension incidence and associated factors in healthy parturients undergoing cesarean delivery after neuraxial anesthesia.^8^ However, parturients with COVID-19 have not been included in those studies.

The present study was designed to evaluate the effects of NLA during labor in patients with COVID-19. The frequency and safety of NLA regarding hemodynamic stability and neurological complications were evaluated to identify any factors that may influence its use in daily clinical practice.

## Methods

This nationwide retrospective, observational, controlled study included patients enrolled in the Qatar precious register system (Q-PRECIOUS register). This consultant-led research platform gathers information from maternity hospitals in Qatar about certain pregnancy-related diseases and complications in the national population. Selected physicians at each center were asked to enroll COVID-19-positive parturients admitted to their hospitals in labor and received NLA from March 1, 2020, to June 30, 2020. This cohort was compared with a control group consisting of an equal number of randomly selected noninfected women in labor who received NLA from March 1, 2019, to June 30, 2019. Inclusion criteria were COVID-19 positive and in labor for normal delivery under neuraxial techniques. Exclusion criteria were failed epidural/combined spinal-epidural (defined by incomplete/partial block).

Confirmed maternal infection was defined as the presence of viral RNA on polymerase chain reaction (PCR) testing of nasopharyngeal swabs. From March 1 to May 1, 2020, women were tested only if they were symptomatic, were in contact with a positive family member, or had traveled abroad during the previous two weeks. After May 1, 2020, women in labor with or without symptoms of COVID-19 infection were routinely tested upon hospital admission. Neonatal infection was defined as PCR detection of viral RNA in a nasopharyngeal swab or aspirate. Data were collected by obstetric anesthetists. The study protocol was approved by Hamad Medical Corporation (HMC), Medical Research Center (MRC) MRC-01-20-431, through a fast-track process after being categorized as an urgent public health priority study. Waiver of informed consent has been secured before data collection from HMC-MRC because of the retrospective nature of the study.

For combined spinal-epidural (CSE), initial rapid analgesia was provided by spinal injection of 0.5 ml of 0.5% heavy Marcaine and 25-mcg fentanyl, followed by epidural infusion of a low dose fentanyl and levobupivacaine mixture (0.1% levobupivacaine with fentanyl 2 mcg/ml) through patient-controlled epidural analgesia. For epidural analgesia, the same epidural mixture was used as an infusion. Hypotension was defined as either a reduction of >20% from baseline or systolic blood pressure < 100 mmHg, while tachycardia was defined as heart rate (HR) of more than 100 beats per minute (bpm) and bradycardia HR of < 50 bpm.^9^

Sample characteristics and the distribution of various parameters, including demographic characteristics, signs and symptoms, clinical features, and postoperative outcomes, were summarized by descriptive statistics measures and others. The number of enrollable participants was allocated according to the total case number encountered in the specific study timeline, then assigned a matching number to the control group. Categorical variables were expressed as frequency (percentage), normally distributed continuous variables as mean ±  standard deviation (SD) with corresponding 95% confidence intervals and nonnormally distributed continuous variables as median and interquartile range. A two-sided *p* value < 0.05 was considered statistically significant. All statistical analyses were performed using Statistical Package for the Social Sciences, IBM Corp, Armonk, NY, USA (SPSS) 18.0 statistical software.

## Results

### Demographic characteristics

This study enrolled 32 COVID-19-positive parturients from March 1, 2020, to June 30, 2020, and a matched historical group of 32 COVID-19-negative parturients from March 1, 2019, to June 30, 2019. Of the 32 COVID-19-positive women, 14 (43.8%) were obese [body mass index (BMI) ≥  30], 12 (37.5%) had gestational diabetes (blood glucose ≥  190 mg/dl), five (15.6%) had hypothyroidism [thyroid-stimulating hormone (TSH)>4 mu/l], and two (6.3%) had anemia (Hb < 11 g/dl). In contrast, of the 32 COVID-19 negative women, 19 (59.4%) were obese, seven (21.9%) had gestational diabetes, two (6.3%) had hypothyroidism, and none (0%) had anemia. The course of gestation was uneventful in all 64 patients (Table 1).

**Figure 1.**
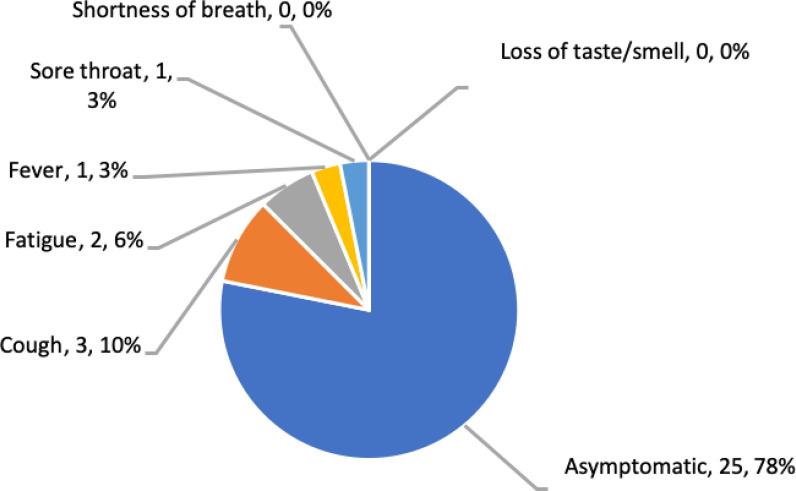
Clinical presentation at the time of delivery in the COVID-19-positive group

Of the 32 patients in the COVID-19 group, 25 (78.1%) were asymptomatic, three (9.3%) had a cough, two (6.3%) had fatigue, one (3.1%) had a low-grade fever (temperature < 39°C), and one (3.1%) had a sore throat (Figure 1). Additionally, one parturient (3.1%) had lymphopenia ( < 1000 cells/μl) and 13 (40.6%) had mildly elevated C-reactive protein concentrations (10 mg/L). All patients had normal liver function tests and renal function tests. All 32 were PCR-positive for SARS-CoV-2 RNA, seven (21.9%) were positive for patchy ground-glass opacities on chest X-rays, and none underwent computed tomography (CT) scanning.

### Obstetric and neonatal outcomes

The 64 mothers in both groups had singleton pregnancies. No adverse neonatal outcomes (neonatal deaths or severe neonatal asphyxia) were observed in either group (Table 2). In the COVID-19 group, 21.9% of neonates were delivered by emergency cesarean section due to acidosis (pH < 7.2), nonreassuring cardiotocography (CTG), and failure to progress, and 18.8% were delivered vaginally with instrument assistance. In the control group, 6.3% of neonates were delivered by emergency cesarean and 6.3% with instrument assistance (Table 2). One neonate in the COVID-19 group had a birthweight of < 2500 g. The Apgar scores of all neonates in both groups ranged from 8 to 9 at 1 minute and 9 to 10 at 5 minutes. Of the 32 neonates in the COVID-19 group, 90.6% were transferred to the COVID-19 ward for close monitoring. All 32 neonates in the COVID-19 group were tested for SARS-CoV-2 RNA using RT-PCR. Two (6.3%) tested positive and were admitted to the neonatal intensive care unit for further care (Table 3). All neonates in both groups were discharged from the hospital in good health. A comparison of the neonates in the two groups showed no clinical differences.

### Anesthesia management and mode of delivery

**Figure 2.**
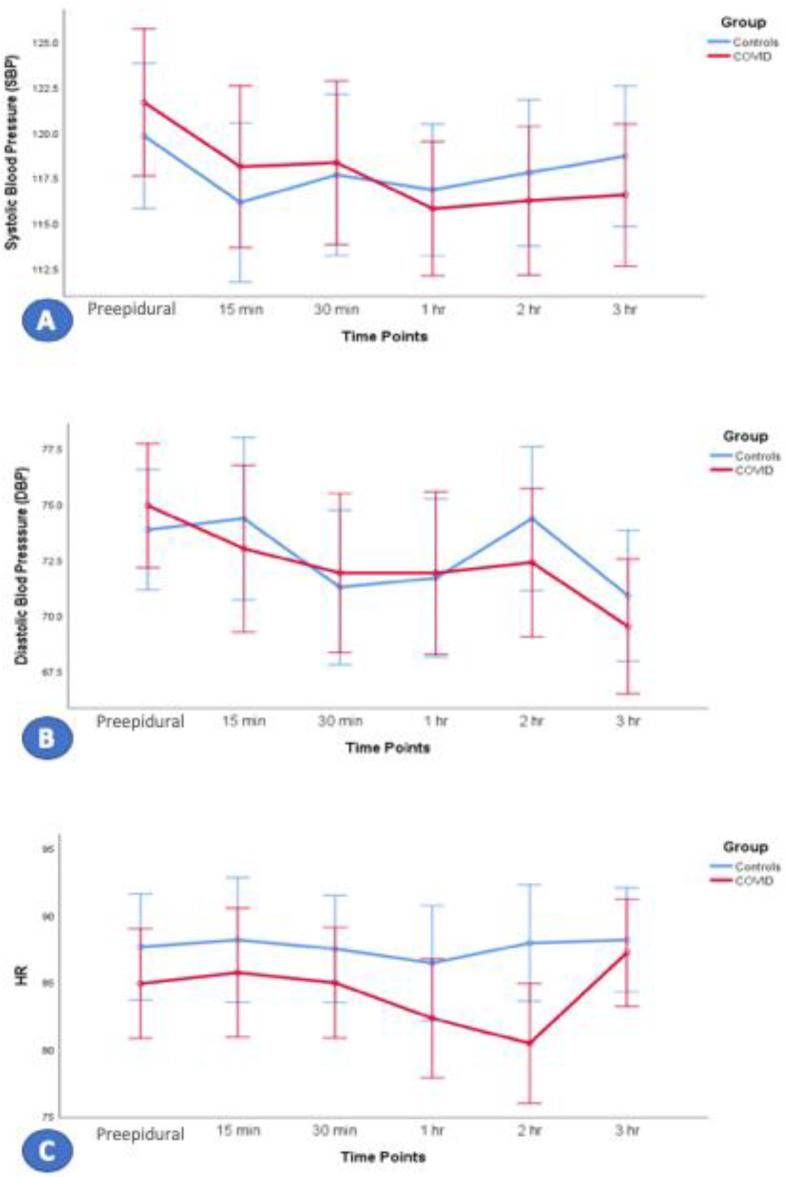
Effects of neuraxial labor analgesia anesthesia over time on (A) systolic blood pressure (BP), (B) diastolic BP, and (C) heart rate (HR) in patients in the COVID-19 and control groups

**Figure 3.**
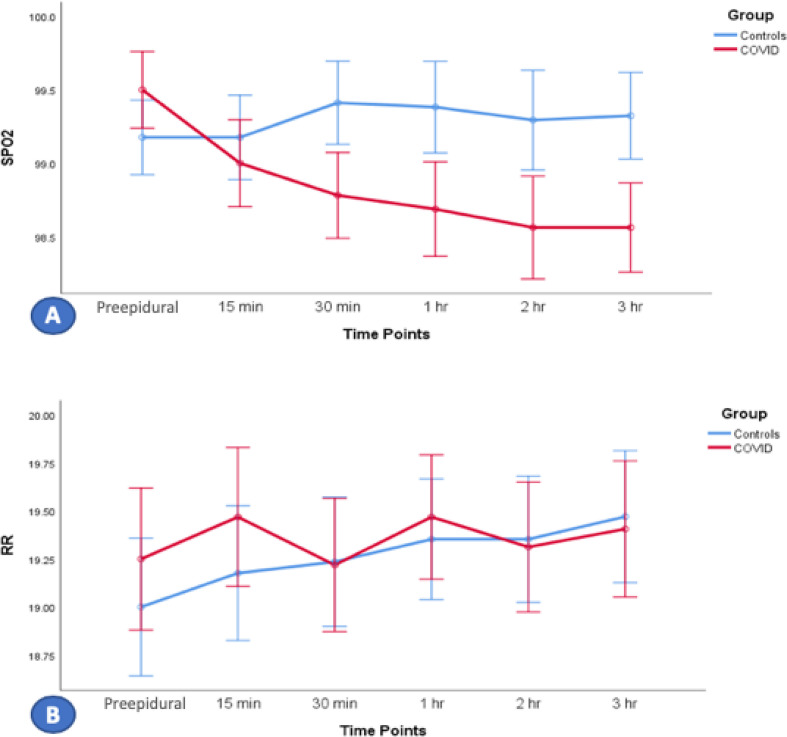
Effects of neuraxial labor analgesia anesthesia over time on oxygen saturation (SPO2) and (B) respiratory rate (RR) in patients in the COVID-19 and control groups

Of the 32 women in the COVID-19 group, 19 (59.4%) underwent normal vaginal delivery, six (18.8%) underwent instrument-assisted vaginal delivery, and seven (21.9%) underwent emergency cesarean section. In the control group, 28 (87.5%) women underwent vaginal delivery, and two (6.3%) each underwent instrument-assisted vaginal delivery and emergency cesarean section. All patients in both groups received NLA. In the COVID-19 group, 24 (75%) received epidural, and eight (25%) received CSE anesthesia, whereas in the control group, 29 (90.6%) received epidural and three (9.4%) received CSE anesthesia. Patients in both groups were hemodynamically stable, as determined by blood pressure, respiratory rate, and oxygen saturation. However, hemodynamic stability was significantly lower in the COVID-19 group than in the control group (Figures 2 and 3). The hemodynamic stability was lower in the COVID-19 group regarding blood pressure (BP), HR, oxygen saturation (SPO_2_), and respiratory rate (RR), although they were in a normal range. This can be explained by vasodilation and heat loss as signs of infection, increased numbers of topups, and the rate of CSE compared with those of epidural in COVID-19 patients, which can induce hypotension, bradycardia, and low SPO_2_ as signs of a high block (hypotension; systolic BP < 100 mmHg, bradycardia; HR < 60 bpm, nausea, dyspnea, and upper extremities weakness). However, additional studies with a higher cohort number are needed to confirm these findings in the future. Patients in the COVID-19 group had a lower mean body temperature than the control group, possibly due to infection-induced peripheral vasodilatation associated with heat loss or the regular use of analgesics with antipyretic effects (4). All patients with COVID-19 recovered entirely and were discharged home after 2-week isolation in the hospital (Table 3).

## Discussion

The present study found that the clinical presentation of COVID-19-positive parturients was atypical, as 78% were asymptomatic when they presented in labor, despite having positive PCR tests for SARS-CoV-2 RNA.^10^ Of the remaining patients, 10% presented with cough, 6% with fatigue, 3% with fever, and 3% with a sore throat. None of these patients were critically ill, hypoxic, or required oxygen supplementation during delivery. Seven patients had abnormal chest X-rays, with all having mild symptoms. A study of large numbers of nonpregnant COVID-19 patients in China found that the general clinical presentation began with fever followed by fatigue and cough, with nausea and vomiting being relatively rare.^3^ Many COVID-19-positive patients experience a sudden loss of smell and taste,^11^ but this was not observed in any of our patients. Many nonspecific symptoms observed in COVID-19-positive parturients were similar to nonaffected women.^12^ For example, nausea and vomiting are common in pregnancy, and parturients may experience vomiting, myalgia, and diarrhea during the latent delivery phase. In contrast, the presentation of several pregnancy-related conditions, such as preeclampsia during pregnancy and delivery, is similar to COVID-19 symptoms, such as headache and shortness of breath. Furthermore, fever is the most prominent presenting symptom in patients with chorioamnionitis, leading obstetricians to overlook COVID-19 infection as a likely diagnosis. Moreover, most COVID-19-positive parturients are asymptomatic and diagnosed only when they present in delivery or after birth.^13^ The asymptomatic nature of their disease can result in inadvertent exposure to their families and healthcare workers. Furthermore, pregnancy does not increase the risk of exposure to severe COVID-19 disease, in as much as the disease has the same clinical course in both pregnant and nonpregnant patients.^14^

Although this study found that normal vaginal delivery with early NLA was safe for parturients with COVID-19, at present, insufficient data have been collected to determine the optimal mode of delivery. Selection of the mode of delivery should be mainly based on parturient condition and wishes. Emergency cesarean section should be considered if there are fetal or maternal indications, and the epidural catheter can be topped up to avoid general anesthesia. In our study, 21.8% of COVID-19-positive parturients required emergency cesarean because of fetal distress and no progress, and 18.7% underwent instrument-assisted vaginal delivery with *p* values of 0.01 and 0.05, respectively. The course of delivery was more complicated in the COVID-19 group, as only 59% were delivered vaginally. The higher rates of instrument-assisted delivery or emergency cesarean section in the COVID-19 group than in the control group may be due to differences in rates of primigravida, which were 46% in the COVID-19 group and 15% in the control group (*p* < 0.01). Similarly, the significantly longer duration of delivery and the significantly greater total blood loss in the COVID-19 group may also be because of the higher rate of a primigravida in this group.

High maternal and fetal metabolic needs during pregnancy and delivery result in hemodynamic changes.^15^ High cardiac output, expanded plasma volume, decreased vascular resistance, and enlargement of the gravid uterus compressing the inferior vena cava significantly reduce blood return to the heart. Moreover, blockage of the sympathetic nervous system induced by NLA and subsequent arterial and venous vasodilation and functional hypovolemia are confounding responses to these physiological changes, greater in the COVID-19 group than in the control group. Greater hemodynamic changes and reduced rate of hemodynamic stability during delivery in the COVID-19 group might be explained by the longer duration of delivery with epidural analgesia, the higher incidence of CSE (25% vs. 9.4%) (*p* < 0.05), the higher estimated blood loss requiring multiple uterotonic medications (37.5% vs. 9.4%) (*p* < 0.05), and the higher rate of operative deliveries in the COVID-19 group than in the control group. Our findings also agreed with the higher incidence of hypotension in women in the COVID-19 group who delivered by cesarean section while under epidural than general anesthesia, which may warrant close monitoring and observation.^16^ None of the patients in either group required vasopressor or oxygen supplementation during delivery, and there were no other differences in clinical parameters between the two groups.

Before NLA placement, a complete blood count should be determined in COVID-19 positive parturients. Although a study in China found that thrombocytopenia was present in COVID-19-positive patients,^17^ none of the patients in the present study had significant hematological abnormalities, with only one having mild thrombocytopenia. Seeding the subarachnoid space with viremic blood could result in encephalitis or meningitis, but this was not observed in the present study nor in any previous study of patients who had undergone NLA.^18^ Only one patient in the COVID-19 group experienced a postdural puncture headache (PDPH), which responded well to conservative management. Otherwise, there were no differences in neurological complications between the COVID-19 and control groups.

Because the virus mainly targets the lungs, the selection of analgesic technique is vital for COVID-19-positive women in labor. The absence of epidural anesthesia may increase the possibility of conversion to general anesthesia if an emergency cesarean section is required. Labor pains induce physiological changes that can affect both maternal and fetal welfare. Severe pain primarily affects the respiratory system by inducing hyperventilation and subsequent respiratory alkalosis, resulting in a leftward diversion of the oxyhemoglobin dissociation curve and decreased oxygen delivery to the fetus.^19^ Pain, tension, and nervousness enhance the concentrations of circulating cortisol, catecholamines, and adrenaline, disturbing uterine contractions and uteroplacental blood flow, which can be altered by satisfactory analgesia during labor.^19,20^ Furthermore, postpartum depression is more frequent among women who deliver without any analgesic, with the intensity of labor pains positively associated with posttraumatic stress syndrome.^20^ Although this has increased the frequency of use of NLA,^21^ relatively little is known about NLA in COVID-19 infection and the effect of NLA on COVID-19 treatment during pregnancy and delivery. Similar to findings in patients with SARS, pneumonia during pregnancy has been associated with maternal and fetal adverse outcomes.^22^ These findings suggest that, in the absence of contraindications, NLA is recommended in COVID-19 infected parturients as it reduces the worsening of respiratory function during labor and delivery.

In the present study, all women in both groups received NLA. However, more manual rescue topup doses were given to patients in the COVID-19 group than in the control group. This difference may have been due to the higher percentage of primigravida, the longer duration of labor, and the higher operative delivery rate in the COVID-19 group. No differences between the two groups in the quality of analgesia, the degree of sensory and motor block, block-level, and starting time of analgesia were observed (Table 4). However, we observed subnormal hemodynamic changes in the COVID-19 group, with BP, oxygen saturation, and HR reduced but still within normal ranges in the COVID-19 group than in the control group. None of these patients required vasopressor or oxygen supplementation, and none experienced end-organ damage.

Two (6.25%) neonates in our study were positive for COVID-19, one was 24 hours old, and the other was 36 hours old, suggesting the possibility of vertical transmission of the virus. This finding is consistent with the results of a systematic review and metaanalysis from China, which found that the proportion of infected neonates was low (6%), indicating a low but finite possibility of vertical transmission.^23^ We also found that the incidence and severity of COVID-19 disease were lower in neonates than in adults, similar to findings during the SARS epidemic.^12^ As these findings suggest a low but definitive potential of vertical transmission of coronavirus, appropriate handling at birth and early separation of the neonate following delivery are considered essential measures to reduce the incidence of vertical transmission. The findings of this study have to be seen in the light of two limitations that could be addressed in future research, including the relatively small number of patients and the lack of previous research studies on the topic to compare our findings.

## Conclusion

Most COVID-19-positive parturients in labor were asymptomatic, with most testing positive during routine screening. The disease course in these women was mild and benign. None experienced any hematological abnormalities, such as thrombocytopenia. Hemodynamic stability was lower in the COVID-19 group than in a group of noninfected parturients, emphasizing the importance of close monitoring and judicious management. No adverse maternal or neonatal outcomes were observed. All mothers were discharged without any significant complications. There was no statistically significant difference in neurological complications rate in COVID-19 and uninfected parturients. There was no observed difference in the quality of analgesia, degree of sensory and motor block, block levels, and time of starting analgesia in both groups, which could be investigated in future research investigations. More studies are needed to explain the possible effects of COVID-19 infection on parturients receiving NLA.

### Acknowledgments

No external funding was received for the development of this article.

### Funding

No external funding was received for the development of this article.

### Financial Disclosures

The authors report no conflicts of interest relevant to the contents of this article.

## Figures and Tables

**Figure 4.**
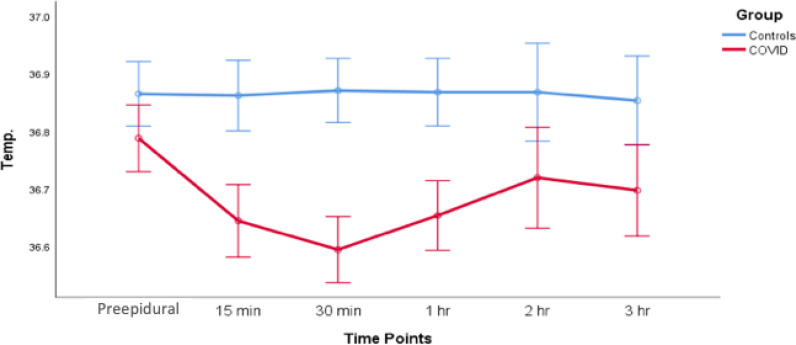
Effects of neuraxial labor analgesia anesthesia over time on body temperature in patients in the COVID-19 and control groups

**Table 1:** Demographic characteristics of women with and without COVID-19

		COVID-19 positive	Control

	Number	32	32

Age (year)	< 20	0	0

	20–34	26	12

	>35	6	20

BMI (kg/m^2^)	Normal < 25	5	4

	Overweight 25–30	13	9

	Obese >30	14	19

Coexisting morbidities	Thyroid disorders	5	2

	Gestational diabetes	12	7

	Anemia	8	0

	Others	3	0


**Table 2:** Pregnancy outcomes of women in the COVID-19 and control groups.

		COVID-19 positive	Control	*p* value

Primigravida		15	5	

Multigravida		17	27	

Gestational age (weeks)	< 37	0	2	

	>37	32	30	

Mode of delivery	Normal	19	28	0.005

	Instrumental	6	2	0.05

	Emergency lower segment cesarean section	7	2	0.01

Uterotonics	Single medication	20	29	0.05

	Multiple medications	12	3	0.002

Mean duration of labor (min)	Mean ± SD	490.3 ± 72.3	384.65 ± 66.6	0.5

Blood loss (ml)	Mean ± SD	406.4 ± 102	195.62 ± 32.1	0.005

	< 500	27	32	0.01

	>500	3	0	0.1

	>1000	2	0	0.1

Treatment	Antiviral	0		

	OH chloroquine	7		

	Steroids	0		

	Antibiotics	8		

	Others (vitamin C/plasma)	2		

Discharge		32	32	

Death		0	0	


COVID 19; coronavirus disease. EM LSCS; emergency lower segmental cesarean section. SD; standard deviation

**Table 3:** Neonatal outcomes, including COVID-19 status, Apgar scores, and birth weights, in the COVID-19 and control groups

		COVID-19 positive	Control

Apgar	1 min (score 8– 9)	32	32

	5 min (score 9–10)	32	32

Birth weight (kg)	< 2.5	1	0

	>2.5	31	32

Acidosis	pH < 7.2	3	0

Clinical outcomes	Neonatal ICU admission	3	0

	Discharged from hospital	32	32

	Death	0	0

Asphyxia		0	0

COVID-19 positivity		2	0


COVID 19; coronavirus disease

**Table 4:** Anesthesia outcomes in patients in the COVID-19 and control groups. Combined spinal-epidural, postdural puncture headache, American Society of Anesthesiologists

	COVID-19 positive	Control	*p* value	

American Society of Anesthesiologists	ASA II	30	32	

	ASA III	2	0	

Entonox		5	6	0.2

Morphine		0	6	0.06

Neuraxial	Epidural	24	29	0.05

	CSE	8	3	0.005

Cervical dilatation	< 4 cm	7	6	

	>4 cm	25	26	

Number of topups		9	2	0.003

Vasopressors		0	0	

Complications		1 (PDPH)	0	


CSE; Combined Spinal Epidural, PDPH; Post Dural Puncture Headache

